# The Cue-Response Mental Link: Its Critical Role in the Downregulation of Disgust by Perspective Taking Implementation Intention

**DOI:** 10.1177/00332941241291030

**Published:** 2024-10-06

**Authors:** Patrick Gomez

**Affiliations:** Department of Occupational and Environmental Health, 30678 Unisanté, Center for Primary Care and Public Health & University of Lausanne, Lausanne, Switzerland

**Keywords:** Implementation intentions, emotion regulation, reappraisal via perspective taking, disgust, goal setting, affective pictures

## Abstract

Implementation intentions are if-then plans that create a mental link between a situational cue and a goal-directed response that people can form to help them achieve emotion regulation goals more effectively. The main goal of this study was to determine if forming the goal intention to not get disgusted together with a perspective taking implementation intention is more effective than forming the goal intention to not get disgusted that spells out the same perspective taking strategy but without linking it to the cue. Eighty-six female participants viewed disgusting, neutral, and pleasant pictures under four instructions: no emotion regulation instructions (CG), the goal intention to not get disgusted (GI), this goal intention furnished with the perspective taking regulation strategy (GI-PT), and this goal intention in tandem with the perspective taking implementation intention (PT-II). Compared with CG, GI, and GI-PT participants, PT-II participants showed a significantly larger decrease in disgust when seeing the disgusting pictures. This effect remained constant across repeated exposure to the critical contents and was larger among individuals who did not consciously try to reappraise the disgusting pictures than among individuals who consciously tried to reappraise them. Valence rating, arousal rating, and sympathetic activity did not significantly differ between conditions. We conclude that it is the if(situational cue)-then(goal-directed response) link created by forming the perspective taking implementation intention that accounts for the positive effect on disgust and not simply the information about the perspective taking behavior to adopt.

## Introduction

Despite feeling committed to attain desired end states, people often struggle to translate their goal intentions (“I want to reach Z!”) into appropriate behaviors (Webb & Sheeran, 2006). Implementation intentions (Gollwitzer, 1999) are plans that people can form to help them realize their goal intentions. They typically have an if-then structure (“If situation X is encountered, then I will perform behavior Y!”), which allows people to create a mental link between a critical situational cue (expressed in the if-part) with a goal-directed response (expressed in the then-part). The activation of the mental representation of the critical situational cue and the formation of the cue-response mental link are supposed to change the response control from effortful and conscious to effortless and automated (Brandstätter et al., 2001; Gollwitzer & Schaal, 1998; Parks-Stamm et al., 2007; Webb et al., 2012a; Webb & Sheeran, 2007, 2008). Implementation intentions have been found to help different types of populations achieve their goals in numerous domains (e.g., Brewster et al., 2015; Gollwitzer & Sheeran, 2006; Hall et al., 2014; Stalbovs et al., 2015; Stern & West, 2014; Tanis et al., 2023), including emotion regulation goals (Webb et al. 2012a).

Emotion regulation “refers to attempts to influence which emotions one has, when one has them, and how one experiences or expresses these emotions” (Gross, 2015, pp. 4–5). Perspective taking describes an emotion regulation strategy consisting in adopting a specific perspective in order to alter the effect of an affective stimulus or situation (Webb et al., 2012b). For example, individuals can view events from a self-distanced, third-person perspective (e.g., Ayduk & Kross, 2008; Ochsner et al., 2004). Perspective taking is aimed at activating alternative meanings of the affective stimulus or situation at hand. As such, perspective taking can be classified as belonging to the cognitive change family, one of the five families of the process model of emotion regulation by Gross (2015). Meta-analytic analyses have shown that perspective taking is one of the most effective ways of changing emotions at the experiential level (i.e., self-reported emotions/feelings; Webb et al., 2012b).

The downregulation of negative emotions, in particular unpleasant feelings, is the most common emotion regulation effort (Gross et al., 2006). One negative emotion is disgust. Disgust derives from the Latin dis- (expressing reversal) and gustus (taste) and describes in lay terms a strong feeling of distaste, revulsion, dislike, or disapproval. Disgust can be seen as a rejection response aimed at protecting the individual from contaminants belonging to a broad range of categories including for instance food, body products, animals, sexual behaviors, and contact with death or corpses (Rozin et al., 2008). Thus, different types of disgust can be distinguished. Disgust is part of numerous psychiatric conditions (Rozin et al., 2008), and its appropriate regulation is a necessity in many life and work settings (Diefendorff et al., 2008).

Research on emotion regulation by implementation intentions has grown in the last 10–15 years. Overall, it is suggested that forming implementation intentions is helpful for enacting desired emotion regulation strategies to regulate emotions such as anger (Schweiger Gallo et al., 2018), fear (e.g., Azbel-Jackson et al., 2016), grima (Schweiger Gallo et al., 2017), and disgust (e.g., Hallam et al., 2015). The focus of the present study is the downregulation of disgust that is experienced in relation to mutilation, injury, and blood. Compared with both a goal intention only condition and a no goal intention condition, the goal intention ‘‘I will not get disgusted!’’ followed by the perspective-taking implementation intention ‘‘And if I see blood, then I will take the perspective of a physician!’’ significantly decreased unpleasantness reported after seeing images showing bloody bodies presented for 100 ms (Gallo et al., 2012). Using a pre-post study design, Gomez et al. (2015) extended this finding by showing that forming the same perspective taking implementation intention significantly reduced unpleasantness and disgust feelings to the same disgusting pictures presented for 6 s significantly more than forming the mere goal intention to not get disgusted and without inducing increased sympathetic activation during the entire emotion regulation task. More recently, Chen et al. (2020) found that the same implementation intention reduced unpleasantness of both low- and high-intensity disgusting pictures compared with a control condition. This study did not include a goal intention condition. While Gallo et al. (2012) and Gomez et al. (2015) reported no significant effects of the perspective taking implementation intention on arousal rating, Huang et al. (2020) found that the same implementation intention significantly reduced not only unpleasantness but also arousal compared with both goal intention and no goal intention.

The present study aims at advancing the field of emotion regulation via implementation intentions by addressing three important questions and limitations. First, the effectiveness of implementation intentions for regulating emotions has been investigated by comparing mere goal intention conditions with goal intention plus implementation intention conditions. It could be argued that the latter is more helpful because participants are given more information about what to do to reach their emotion regulation goal. There is evidence that the higher effectiveness of a goal intention plus implementation intention condition remains when the goal intention includes the information on the behavior of the implementation intention in problem solving (Bayer & Gollwitzer, 2007). We aim to test if this also holds for emotion regulation tasks by including a condition that spells out the then-part of the implementation intention but without linking it to the cue of the if-part.

A second important question is whether the effectiveness of implementation intentions in regulating emotions varies across repeated exposure to the target stimuli. As suggested by Adriaanse et al. (2011), if the behavior of the then-part is repeatedly chosen in the critical situation, the mental link between the situation and the behavior might be strengthened over time. Thus, it might be predicted that individuals being instructed to form an implementation intention to regulate their emotions become better at reaching their emotion regulation goal across repeated exposure to the target stimuli.

Third, researchers conducting studies on emotion regulation and implementation intentions have used traditional statistical approaches such as ANOVA and ANCOVA (e.g., Azbel-Jackson et al., 2016; Gomez et al., 2015). With these methods, responses are aggregated across trials. By doing so, important information about variance in the data is lost. By avoiding data aggregation, multilevel mixed-effects modeling allows for more accurate data structure specification and effect estimation (Baayen et al., 2008; West et al., 2022). Data aggregation can lead to significant bias when the sphericity assumption is violated and when item variance is present in addition to person variance (Schad et al., 2024). With multilevel mixed-effects modelling on unaggregated data, the full random effects structure can be explicitly modelled, i.e., both between-persons variance and between-items variance in the dependent variables are taken into account.

In sum, we aimed to test the following hypotheses. First, forming the goal intention to not get disgusted in combination with the perspective taking implementation intention was expected to reduce self-reported disgust and unpleasantness in response to disgusting contents significantly more than forming (1) no emotion regulation goals, (2) the mere goal intention to not get disgusted, and (3) the goal intention to not get disgusted that spells out the perspective taking strategy of the then-part of the implementation intention but without linking it to the cue of the if-part. Second, the effect of receiving the goal intention to not get disgusted in combination with the perspective taking implementation intention was predicted to become larger across repeated exposure to the disgusting contents, relative to the other three conditions. Given mixed findings in the literature, no hypothesis was formulated for the effect of the emotion regulation instructions on arousal rating of the disgusting contents. Finally, sympathetic activation indexed by skin conductance level (SCL) during the entire emotion regulation task was expected to not significantly vary as a function of whether participants form or not the goal intention to not get disgusted in combination with the perspective taking implementation intention^1^.

## Method

### Participants

Relying on our experience (Gomez et al., 2015), we anticipated that it would be very difficult to recruit an equal number of male and female participants. Therefore, we recruited only female participants to eliminate a source of variability. Eighty-eight young women with French mother tongue and good self-reported general health participated. Two participants stopped the experiment before the presentation of the second set of pictures, and their data were excluded from all analyses. The remaining 86 women completed the entire experiment. Table 1 provides additional information about the participants. We determined the sample size considering the sample size of similar studies in the field (Azbel-Jackson et al., 2016; Gallo et al., 2012; Gomez et al., 2015; Schweiger Gallo et al., 2017). The study was approved by the Human Research Ethics Committee of the canton of Vaud, Switzerland.

**Table 1.** Participants’ Characteristics and Responses to the Post-presentation Questionnaire About Commitment, Emotion Regulation Strategies, and Perceived Performance by Experimental Group (*M*s and *SD*s in Parentheses).

	CG	GI	GI-PT	PT-II	*F* statistics	*p* value	η_p_^2^
	(*n* = 21)	(*n* = 21)	(*n* = 23)	(*n* = 21)			
Age (years)	26.9 (8.4)	24.8 (6.7)	26.5 (5.7)	25.9 (6.7)	*F*(3, 82) = 0.38	0.77	0.01
Disgust sensitivity	1.94 (0.37)	2.04 (0.45)	2.06 (0.51)	1.96 (0.37)	*F*(3, 82) = 0.40	0.75	0.01
Habitual use of cognitive reappraisal	5.01 (0.77)	4.50 (1.08)	4.70 (1.03)	5.06 (1.14)	*F*(3, 82) = 1.44	0.24	0.05
Commitment	8.19 (0.81)	8.00 (1.48)	7.96 (1.55)	7.90 (1.55)	*F*(3, 82) = 0.22	0.88	0.01
Control of negative feelings	3.62 (2.38)	5.81 (2.71)	6.26 (2.22)	7.05 (1.88)	*F*(3, 82) = 8.49	<.001	0.24
Reappraisal of unpleasant pictures	4.57 (2.46)	6.00 (2.51)	6.09 (2.56)	7.19 (2.06)	*F*(3, 82) = 4.17	0.008	0.13
Ignoring unpleasant pictures	3.43 (3.07)	4.00 (2.45)	3.30 (2.93)	4.57 (2.64)	*F*(3, 82) = 0.94	0.43	0.03
Difficulty controlling negative feelings	n/a	5.52 (2.46)	6.30 (2.36)	6.10 (2.57)	*F*(2, 62) = 0.58	0.56	0.02
Usefulness of instructions	n/a	5.10 (2.68)	4.52 (3.09)	6.62 (2.01)	*F*(2, 62) = 3.63	0.032	0.11
Success in reaching instruction’s goal	n/a	6.14 (1.46)	6.13 (2.30)	6.19 (1.78)	*F*(2, 62) = 0.01	0.99	0.00

Note. CG = control group (no emotion regulation instructions); GI = goal intention group; GI-PT = goal intention with perspective taking strategy group; PT-II = goal intention with perspective taking implementation intention group.

### Design

The design of this study was a 4 (Experimental condition) × 3 (Picture type) × 2 (Picture set) factorial design. Experimental condition is a between-subjects factor with the group CG receiving no emotion regulation goals, the group GI receiving the goal intention to not get disgusted, the group GI-PT receiving the goal intention to not get disgusted together with the perspective taking behavior spelled out, and the group PT-II receiving the goal intention to not get disgusted with the perspective taking implementation intention. Picture type is a within-subjects factor with the three categories of disgusting, neutral, and pleasant pictures. Picture set is a within-subjects factor with Set 1 and Set 2. Moreover, to address our second hypothesis, Set 2 was divided into three blocks, each consisting of the same number of pictures.

### Stimuli

We used 68 pictures of the International Affective Picture System (IAPS; Lang et al., 2005; see the Supplemental material for the IAPS numbers and the normative valence and arousal ratings). At the start of the experiment, we showed six pictures to familiarize the participants with the procedure. The other 62 images were divided into Set 1 with 16 images and Set 2 with 46 images. The first image of both sets was affectively neutral and was used as ‘‘filler’’ and not analyzed. The remaining images of Set 1 were five disgusting, five neutral, and five pleasant pictures, and the remaining images of Set 2 were 15 disgusting, 15 neutral, and 15 pleasant pictures.

We used E-prime 2.0 Professional to present the pictures on a 19 in. computer screen situated at 60–70 cm from the participants’ eyes. The pictures were shown for 6 s with an intertrial interval of 22–26 s in five orders that were counterbalanced across experimental conditions. To assure an even distribution of the three picture categories, each block of three pictures included one disgusting, one neutral, and one pleasant image. Across the five presentation orders, the same image was shown on average in the first, middle, and final part of the set of pictures.

### Measurements

All instructions and questionnaires were administered in paper-and-pencil format.

#### Affective Ratings of the Pictures

We assessed *disgust rating* using a 9-point scale with the anchors *not at all disgusted* and *extremely disgusted* and *valence rating* and *arousal rating* using the 9-point Self-Assessment Manikin (Lang et al., 2005). We scored the three ratings so that 1 corresponds to *not at all disgusted*, *very unpleasant*, and *very low arousal*, and 9 corresponds to *extremely disgusted*, *very pleasant*, and *very high arousal*.

#### Electrodermal Activity

We measured skin conductance with the Psylab device (Contact Precision Instruments, London, UK). Two 8-mm diameter Ag/AgCl electrodes were filled with TD-246 Skin Resistance–Skin Conductance Electrode Paste (Med Associates Inc., St. Albans, VT) and placed adjacently on the hypothenar eminence of the left palmar surface.

#### Post-Presentation Questionnaire

At the end of the picture presentation, participants answered seven questions referring to the presentation of the second set of pictures using 9-point scales ranging from 1 (*not at all*) to 9 (*very*). We assessed *commitment to the instructions* with ‘‘How committed did you feel to the regulation intention/instructions?’’, *emotion regulation* with three questions, ‘‘How much did you try to control your negative feelings when looking at the unpleasant pictures?’’, ‘‘How much did you try to think about the unpleasant pictures in a way that decreased your emotion?’’, and ‘‘How much did you try to ignore the unpleasant pictures?’’, and *perceived performance* with three questions, ‘‘How difficult was it to control negative feelings when looking at the unpleasant pictures?’’, ‘‘Did your regulation intention/instructions help you control negative feelings when looking at the unpleasant pictures?’’, and ‘‘How well did you succeed in realizing the goal expressed in the instructions?’’.

#### Participants’ Characteristics

We assessed two personal characteristics susceptible of modulating emotional reactivity and regulation. We used the 27-item Disgust Scale—Revised (Haidt et al., 1994, modified by Olatunji et al., 2007, example item ‘‘It would bother me tremendously to touch a dead body’’, scale min = 0, max = 4) to measure *disgust sensitivity*. We used the Emotion Regulation Questionnaire (Gross & John, 2003) to measure the *habitual use of cognitive reappraisal* (6 items, example item ‘‘I control my emotions by changing the way I think about the situation I’m in’’, scale min = 1, max = 7).

### Procedure

Participants were tested individually during one session lasting approximately 1 hr. First, the participants were provided with an explanation of the experiment and measurements and signed the informed consent form. They were told that they would look at several images on a computer screen and report their emotions immediately after each image, using three scales for valence, arousal, and disgust. They were asked to perform the ratings spontaneously and quickly. After attaching the skin conductance electrodes, the six practice trials were carried out. Immediately prior to the presentation of Set 1, all participants received the same written instruction: ‘‘We will now show you about fifteen different images. Please, look at all the pictures and rate immediately after each one how you felt during the presentation, using the three corresponding scales’’. After looking at and rating the 16 pictures of Set 1, participants were randomly assigned to one of four conditions. CG participants (*n* = 21) received the same instruction as for the first set of pictures (‘‘fifteen’’ was replaced with ‘‘fifty’’). GI participants (*n* = 21) were asked to form the goal intention ‘‘I will not get disgusted!’’. GI-PT participants (*n* = 23) received an instruction that combined the goal intention and the goal-directed behavior specified in the implementation intention (take the perspective of a physician) but did not mention the critical cue (blood), “In order not to get disgusted, I will take the perspective of a physician!”. PT-II participants (*n* = 21) were asked to form the goal intention ‘‘I will not get disgusted!’’ followed by the implementation intention ‘‘and if I see blood, then I will take the perspective of a physician!’’. Participants were invited to read the instructions and repeat them to themselves until they felt ready. The instruction sheet was handed to the participants inside an envelope, and its content remained unknown to the experimenter throughout the session. Next, Set 2 with 46 images was shown. After the last rating, the experimenter removed the electrodes, and participants completed the questionnaires described above. Finally, the experimenter debriefed the participants about the purpose of the experiment and gave a compensation of 20 Swiss francs.

### Data Reduction and Analysis

Data were complete for all participants. The analyses were performed using SPSS Statistics version 27 (IBM Corp., Armonk, NY, USA).

The three dependent variables valence rating, arousal rating, and disgust rating were analyzed using linear mixed models (LMM) with restricted marginal maximum likelihood estimation (REML). The specified model in terms of fixed effects was the same for the three ratings. It included the fixed main effects and the two-way and three-way interactions for the factors Experimental condition (CG, GI, GI-PT, PT-II), Picture type (disgusting, neutral, pleasant), and Picture set (Set 1, Set 2). As for the random part, we specified crossed random effects associated with each level of participants and pictures. This allowed us to simultaneously account for between-subjects variance and between-pictures variance in the dependent variables (Baayen et al., 2008; West et al., 2022). We tested both random intercepts and random coefficients associated with the various levels of the two crossed random factors and kept them in the model when they explained significant portions of the between-subjects and between-pictures variance. The final models were those with the lowest Schwarz’s Bayesian information criterion. Given the objective and hypotheses of the study, the focus of these analyses was on the three-way interaction. A significant three-way interaction may indicate that the groups differed in the change from Set 1 to Set 2 in their affective ratings of the disgusting pictures. To determine this, a significant three-way interaction was followed up by three contrasts of the estimated changes from Set 1 to Set 2 in the valence, arousal, and disgust ratings of the disgusting pictures comparing PT-II with the other three groups. The same three contrasts were also carried out for the ratings of the neutral and pleasant pictures. Because of the unidirectionality of our hypotheses about the differences between groups, one-tailed comparisons were performed. To counteract the problem of multiple comparisons, we report Bonferroni-corrected *p* values. If the three-way interaction was not significant, we concluded that the groups did not significantly differ in their responses as a function of Picture type and Picture set and, thus, did not perform any contrast analyses. The SPSS syntax of the final LMMs is given in the Supplemental material. To test the robustness of the results of these analyses, we carried out sensitivity analyses by adding participants’ personal characteristics (age, disgust sensitivity, habitual use of cognitive reappraisal), their answers to the post-presentation questionnaire, and presentation order to the models.

To address the hypothesis about the change in affective ratings across repeated exposure to the target stimuli, we ran similar LMMs for the affective ratings of the pictures of Set 2 only. We defined the factor Block with the three levels 1, 2, and 3 referring to the first 15 pictures, the second 15 pictures, and the final 15 pictures, respectively. The specified model in terms of fixed effects included the main effects and the two-way and three-way interactions for the factors Experimental condition (CG, GI, GI-PT, PT-II), Picture type (disgusting, neutral, pleasant), and Block (1, 2, 3). The same procedure as described above was used to specify the random part of the models. The focus of these analyses was also on the three-way interaction. A significant three-way interaction may indicate that the groups differed in the affective ratings of the disgusting pictures across blocks.

For the analysis of SCL, we first calculated the mean skin conductance for the 8-min presentation of Set 1 and the 23-min presentation of Set 2 and then performed LMM with REML including a random intercept for participants and the main fixed effects of Experimental condition and Picture set and their interaction (see Supplemental material for the SPSS syntax).

We used one-way (Experimental condition) ANOVAs to test differences in age, disgust sensitivity, habitual use of cognitive reappraisal, and the seven post-presentation questions. For the three questions related to perceived performance, we only included the three conditions GI, GI-PT, and PT-II.

## Results

### Valence Rating

**Figure 1.**
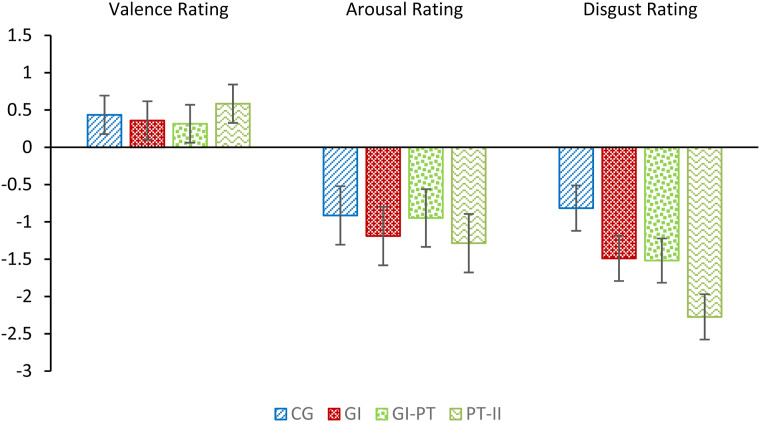
Estimated mean changes from Set 1 to Set 2 in valence rating, arousal rating, and disgust rating of the disgusting pictures for the four experimental conditions. Bars represent *SE*s. CG = control group (no emotion regulation instructions); GI = goal intention group; GI-PT = goal intention with perspective taking strategy group; PT-II = goal intention with perspective taking implementation intention group.

The three-way interaction involving Experimental condition, Picture type, and Picture set was not significant, *F*(6, 257.22) = 0.66, *p* = .68. The main effect of Experimental condition was also nonsignificant, *F*(3, 257.22) = 0.09, *p* = .96, as well as its two-way interactions with Picture type, *F*(6, 257.22) = 0.49, *p* = .81, and Picture set, *F*(3, 257.22) = 0.27, *p* = .85. Figure 1 shows the estimated mean changes from Set 1 to Set 2 in valence rating of the disgusting pictures.

### Arousal Rating

The three-way interaction involving Experimental condition, Picture type, and Picture set was not significant, *F*(6, 4754.02) = 1.27, *p* = .27. The main effect of Experimental condition was also nonsignificant, *F*(3, 82.41) = 0.66, *p* = .58, as well as its two-way interactions with Picture type, *F*(6, 173.24) = 0.28, *p* = .95, and Picture set, *F*(3, 82.41) = 0.35, *p* = .79. Figure 1 shows the estimated mean changes from Set 1 to Set 2 in arousal rating of the disgusting pictures.

### Disgust Rating

The three-way interaction involving Experimental condition, Picture type, and Picture set was significant, *F*(6, 248.16) = 2.34, *p* = .033. The follow-up contrasts showed that PT-II participants had a significantly larger decrease from Set 1 to Set 2 in disgust rating of the disgusting pictures (*M* = −2.27, *SE* = 0.30) than CG participants (*M* = −0.82, *SE* = 0.30; difference PT-II vs. CG: *M* = 1.46, *SE* = 0.31, *p* < .001), GI participants (*M* = −1.49, *SE* = 0.30; difference PT-II vs. GI: *M* = 0.78, *SE* = 0.31, *p* = .017), and GI-PT participants (*M* = −1.52, *SE* = 0.30; difference PT-II vs. GI-PT: *M* = 0.75, *SE* = 0.30, *p* = .019)^2^. Figure 1 shows the estimated mean changes from Set 1 to Set 2 in disgust rating of the disgusting pictures. The four groups did not significantly differ in their change from Set 1 to Set 2 in the disgust rating of the neutral pictures (CG: *M* = 0.38, *SE* = 0.30; GI: *M* = 0.19, *SE* = 0.30; GI-PT: *M* = 0.04, *SE* = 0.30; PT-II: *M* = 0.10, *SE* = 0.30; *p*s > .99) and of the pleasant pictures (CG: *M* = 0.11, *SE* = 0.30; GI: *M* = −0.04, *SE* = 0.30; GI-PT: *M* = 0.13, *SE* = 0.30; PT-II: *M* = 0.14, *SE* = 0.30; *p*s > .99). In the Supplemental material, we report means and *SD*s of the valence, arousal, and disgust ratings for the different experimental conditions, picture types, and picture sets.

### Sensitivity analyses

For valence rating and arousal rating, the three-way interaction Experimental condition by Picture type by Picture set was not significantly modulated by any variable (see Supplemental material).

**Figure 2.**
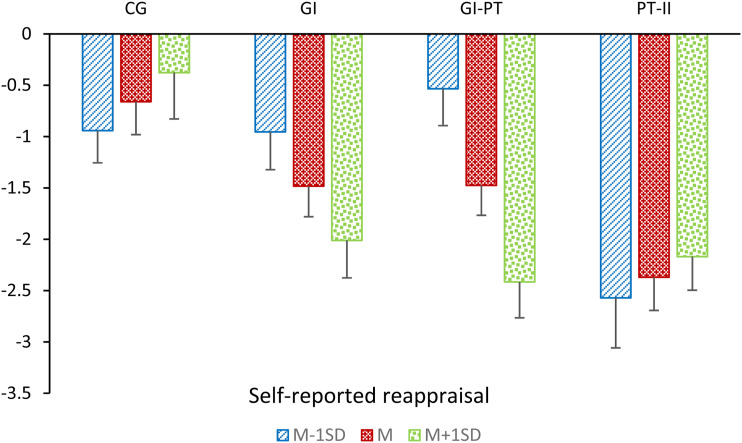
Estimated mean changes from Set 1 to Set 2 in disgust rating of the disgusting pictures for the four experimental conditions as a function of self-reported conscious attempt to reappraise the disgusting pictures (question “How much did you try to think about the unpleasant pictures in a way that decreased your emotion?”, rated on a 1-9 scale). *M* − 1*SD*, *M*, and *M* + 1*SD* correspond to 3.44, 5.97, and 8.49, respectively. Bars represent *SE*s. CG = control group (no emotion regulation instructions); GI = goal intention group; GI-PT = goal intention with perspective taking strategy group; PT-II = goal intention with perspective taking implementation intention group.

For disgust rating, the three-way interaction Experimental condition by Picture type by Picture set was not significantly modulated by any variable except the variable “How much did you try to think about the unpleasant pictures in a way that reduced your emotion?”, *F*(6, 236.07) = 2.36, *p* = .031 (see Supplemental material). To understand this effect, we computed the estimated means of disgust rating for the four groups at three levels of the response scale of the question (*M* – 1*SD =* 3.44, *M =* 5.97, *M* + 1*SD =* 8.49), indicating relatively low, moderate, and high conscious attempt to reappraise the disgusting pictures in a way that reduces the felt emotion, respectively. As can be seen in Figure 2, the mean changes in disgust from Set 1 to Set 2 for the disgusting pictures were different for the four groups across the three levels. For GI and GI-PT participants, as self-reported reappraisal of the disgusting pictures increased, decrease in disgust rating became larger. In contrast, PT-II participants’ decrease in disgust rating of the disgusting pictures varied weakly as a function of self-reported reappraisal of the disgusting pictures. Owing to these different relationships between disgust rating and self-reported reappraisal, PT-II participants reporting relatively low reappraisal of the disgusting pictures had a much larger decrease in disgust rating of the disgusting pictures than their GI and GI-PT counterparts. In contrast, GI, GI-PT, and PT-II participants reporting relatively high reappraisal of the disgusting pictures had similarly large decreases in disgust rating of the disgusting pictures. This response pattern was not observed for disgust rating of the neutral and pleasant pictures (see Figures S1 and S2 in the Supplemental material).

### Affective Ratings Across Blocks of Set 2

The three-way interaction involving Experimental condition, Picture type, and Block was not significant for valence rating, *F*(12, 3548.60) = 0.62, *p* = .83, arousal rating, *F*(12, 3390.85) = 1.09, *p* = .36, and disgust rating, *F*(12, 3547.30) = 0.33, *p* = .98.

### Skin Conductance Level

Descriptive statistics for SCL are reported in Table 2. SCL was not available for one participant in the GI group and for one participant in the GI-PT group. The main effect of Experimental condition was not significant, *F*(3, 80) = 0.29, *p* = .83, as well as the Experimental condition by Set interaction, *F*(3, 80) = 0.22, *p* = .88. In contrast, the main effect of Set was significant, *F*(1, 80) = 22.56, *p* < .001. SCL during Set 2 (*M* = 3.66, *SD* = 1.98) was lower than during Set 1 (*M* = 3.98, *SD* = 1.98).

**Table 2.** *M*s and *SE*s (in Parentheses) for SCL During the Two Picture Sets.

	Set 1	Set 2
CG (*n* = 21)	4.13 (0.46)	3.75 (0.48)
GI (*n* = 20)	4.07 (0.50)	3.69 (0.53)
GI-PT (*n* = 22)	4.13 (0.32)	3.82 (0.33)
PT-II (*n* = 21)	3.59 (0.46)	3.35 (0.40)

Note. CG = control group (no emotion regulation instructions); GI = goal intention group; GI-PT = goal intention with perspective taking strategy group; PT-II = goal intention with perspective taking implementation intention group. SCL is in μS.

### Participants’ Characteristics and Post-Presentation Questionnaire

Statistics for these variables are given in Table 1. There were no significant differences between the four groups for age, disgust sensitivity, and habitual use of cognitive reappraisal.

There were significant group differences for three post-presentation questions. For the question “How much did you try to control your negative feelings when looking at the unpleasant pictures?”, the CG group tried significantly less to control their negative feelings than the GI group, *p* = .012, the GI-PT group, *p* = .001, and the PT-II group, *p* < .001. The three latter groups did not differ significantly from each other, *p*s > .52.

For the questions “How much did you try to think about the unpleasant pictures in a way that reduced your emotion?”, PT-II, GI-PT, and GI participants had higher means than CG participants, with only the difference between PT-II and CG being significant, *p* = .004.

Finally, participants of the three groups receiving self-regulation instructions differed in the perceived helpfulness of the instructions in controlling negative feelings. PT-II was perceived as significantly more helpful than GI-PT, *p* = .033.

## Discussion

In line with our hypotheses, participants who formed the goal intention to not get disgusted in combination with the perspective taking implementation intention (PT-II) reported a larger pre-post decrease in disgust in response to the disgusting pictures than both participants forming the goal intention (GI) and participants forming the goal intention that spells out the perspective taking behavior (GI-PT). Thus, this study extends current knowledge about the effects of using implementation intentions in emotion regulation by showing that the benefits of implementation intentions versus goal intentions do not merely rest on the fact that people using implementation intentions, compared with people who simply set their goals, have additional information about what to do. Bayer and Gollwitzer (2007) provided evidence for this effect in the context of a reasoning task. Participants with a self-efficacy strengthening implementation intention outperformed participants who used a self-efficacy strengthening goal intention. The present study supports the idea, in line with theory (Brandstätter et al., 2001; Gollwitzer & Schaal, 1998; Parks-Stamm et al., 2007; Webb et al., 2012a; Webb & Sheeran, 2007, 2008), that providing a perspective taking emotion regulation strategy in combination with a situational cue in the form of an if-then plan helps participants achieve their emotion regulation goal of decreasing disgust significantly better than providing a perspective taking emotion regulation strategy without linking it to a critical cue.

The sensitivity analyses performed to test possible modulatory effects of participants’ characteristics and behaviors provided another novel finding. The pre-post decrease in disgust rating of the disgusting pictures was significantly modulated by the self-reported attempt to reappraise the disgusting pictures in a way that decreases the emotion. The results illustrated in Figure 2 suggest that PT-II participants were equally successful in decreasing disgust, whether or not they consciously attempted to reappraise the disgusting content. This is in line with the idea that creating the mental link between a critical cue and a goal-directed behavior makes the enactment of the response automatic and unconscious (Brandstätter et al., 2001; Gollwitzer & Schaal, 1998; Parks-Stamm et al., 2007; Webb et al., 2012a; Webb & Sheeran, 2007, 2008). In contrast GI and GI-PT participants were as successful as PT-II participants only for high levels of conscious reappraisal of the disgusting contents. Because the use of reappraisal was assessed after the picture presentation, we cannot exclude that the reported use of reappraisal was the consequence rather than the cause of the disgust ratings. Studies manipulating the conscious use of reappraisal are needed to elucidate this.

Contrary to our hypotheses, the four groups did not significantly differ in their pre-post changes in valence rating of the disgusting pictures. Previous studies have shown the perspective taking implementation intention to decrease unpleasantness when looking at disgusting pictures significantly more than both the control condition and the goal intention condition (Gallo et al., 2012; Gomez et al., 2015; Huang et al., 2020). Gomez et al. (2015) used the same pre-post design as this study and assessed both disgust and valence ratings. Comparing the estimated mean differences between groups observed in these two studies can be informative. In Gomez et al.’s (2015) study, the mean differences between PT-II and CG were 1.84 and 0.68 for disgust and valence, respectively, and the mean differences between PT-II and GI were 1.33 and 0.49 for disgust and valence, respectively. In the present study, the mean differences between PT-II and CG were 1.46 and 0.15 for disgust and valence, respectively, and the mean differences between PT-II and GI were 0.78 and 0.22 for disgust and valence, respectively. Thus, compared with both CG and GI, the effect of PT-II was smaller in the present study than in the previous study for both valence and disgust. What could explain these effect size differences? Methodological aspects are unlikely to be responsible given that the two studies had the same design and materials. Differences in participants’ characteristics or behaviors may be a more likely cause. Because age, disgust sensitivity, habitual use of cognitive reappraisal, commitment to the instructions, and use of emotion regulation strategies were assessed in both studies, we could compare the three groups CG, GI, and PT-II of the two samples regarding these variables. The results of these analyses are reported in the Supplemental material. We found that the two samples significantly differed only in their attempt to reappraise the disgusting pictures in a way that decreased their emotion. Participants of the present study had higher scores than participants of Gomez et al.’s (2015) study. As discussed above, self-reported attempt to reappraise the disgusting pictures modulated the change from Set 1 to Set 2 in disgust rating of the disgusting pictures in a way that with increasing reappraisal, the difference in effectiveness of PT-II versus GI and GI-PT decreased. Thus, one could speculate that the weaker effect of PT-II in reducing disgust in this study than in Gomez et al.’s (2015) study may partly be explained by the difference in the use of conscious reappraisal by the two samples. Studies are needed to test this hypothesis.

The emotion regulation literature has shown that with more regulation attempts, people get better at applying emotion regulation strategies (Webb et al., 2012b). Contrary to our predictions, the effectiveness of the PT-II instruction did not increase across the picture presentation. This would be in agreement with an interpretation that the perspective taking implementation intention helps decrease disgust immediately, and the same level of effectiveness is maintained throughout repeated exposure. A practical implication for future studies in this area is that fewer stimuli could be used, thus reducing participants’ burden.

Increased sympathetic activation as indexed by SCL accompanies self-control effort (e.g., Mehler et al., 2012; Sheppes et al., 2009). SCL during Set 2 was significantly lower than during Set 1. This reflects the often-observed tendency of SCL to decrease over the course of laboratory experiments (Dawson et al., 2016). Importantly and in line with expectations, there were no significant differences in SCL between experimental conditions. This finding is consistent with an interpretation that regulating emotions via implementation intentions is not physiologically taxing. It would be important to investigate whether this also holds for longer emotion regulation tasks.

Like most studies on emotion regulation and implementation intentions (Gallo et al., 2009; Gallo et al., 2012 Study 1; Gomez et al., 2015; Huang et al., 2020; Schweiger Gallo & Gollwitzer, 2007; but see Gallo et al., 2012 Study 2), self-rated commitment to the regulation instructions was high and did not significantly differ between groups. This is consistent with meta-analytic data showing that commitment to the goal intention is not significantly increased by forming implementation intentions (Webb & Sheeran, 2008).

In line with previous findings (Gallo et al., 2012; Gomez et al., 2015), PT-II participants did not significantly differ from GI and GI-PT participants in the perceived difficulty controlling their feelings when seeing disgusting pictures and in the perceived success in reaching the emotion regulation goal. In contrast, the PT-II instruction was rated as significantly more helpful than the GI-PT instruction. The comparison with previous studies suggests that this difference was due to a high score for the PT-II instruction (6.62) rather than a low score for the GI-PT instruction (4.52). In fact, PT-II participants in the studies by Gallo et al. (2012) and Gomez et al. (2015) had mean scores of 4.65 and 4.75, respectively. Because of these scores, one might have expected to observe larger effects of the PT-II instruction in this study compared with previous studies. As discussed above, we rather observed the opposite. We also note that none of the three performance questions significantly modulated the effect of the regulation instructions on the changes in the affective ratings. Overall, these findings lend support to Gollwitzer’s (1993, 1999) assumption that the effects of implementation intentions rest on unconscious processes of which it is difficult to be aware and which are difficult to report accurately.

The findings of this study are qualified by the following limitations. Emotion is a multifaceted phenomenon consisting of several components including experiential, physiological, and behavioral ones (Mauss & Robinson, 2009). The combination of these different components is desirable in future research on emotion regulation and implementation intention to acquire a more comprehensive understanding.

It could be argued that experimenter demand could be an explanation for the observed effects. This can be ruled out based on findings by Gallo et al. (2009), who reported no significant differences in this regard between mere goal intention conditions and goal intention plus implementation intention conditions.

Our knowledge about individual differences in the effects of implementation intentions in emotion regulation is still scarce. The meta-analysis by Webb et al. (2012a) showed that forming implementation intentions was associated with larger effect sizes among people with “psychological problems” than people without “psychological problems” (e.g., participants with high vs. low levels of test anxiety, Parks-Stamm et al., 2010). The exploration of potential modulators presented in this study provides a foundation on which future research could be based. At this stage, the reported results should be interpreted with caution, and specific studies are needed to test modulatory factors. Finally, the sample in this study consisted of relatively affluent, educated, and young women from a highly industrialized western country. Research on gender differences in emotional processing has been explored through various lenses, including biological, socio-cultural, and evolutionary perspectives (Fischer, 2000; Rupp & Wallen, 2008; Troisi, 2001; Wood & Eagly, 2002). Evidence suggests distinct neuropsychophysiological patterns in emotional processing and regulation between men and women (e.g., Bradley et al., 2001; Gomez et al., 2013, 2019; McRae et al., 2008; Nolen-Hoeksema, 2012). Additionally, normal aging is associated with changes in emotional reactivity and regulation (e.g., Burriss et al., 2007; Gomez et al., 2016; Nolen-Hoeksema & Aldao, 2011; Smith et al., 2005). Finally, cultural, racial, and ethnic factors also appear to influence emotional responses and regulation strategies (e.g., Lim, 2016; Matsumoto et al., 2008; Weiss et al., 2022). Therefore, to generalize the findings of this study, research that includes more diverse samples is desirable.

## Conclusion

We have shown that forming the goal to not get disgusted in tandem with a perspective taking implementation intention was significantly more effective in reducing disgust than forming the goal to not get disgusted that spells out the perspective taking behavior. This effect remained constant during repeated exposure to the disgusting contents and was larger among individuals who did not consciously try to reappraise the disgusting pictures than among those who consciously tried to reappraise them. Valence rating, arousal rating, and sympathetic activation were similar in the different experimental conditions.

## Supplemental Material

Supplemental Material - The Cue-Response Mental Link: Its Critical Role in the Downregulation of Disgust by Perspective Taking Implementation Intention Supplemental Material for Impact of The Cue-Response Mental Link: Its Critical Role in the Downregulation of Disgust by Perspective Taking Implementation Intention by Patrick Gomez in Psychological Reports

## Data Availability

The data that support the findings of this study are available from the author upon reasonable request.

## Notes

1. The analysis of skin conductance responses to the single pictures was not a goal of the present study, because the study was not sufficiently powered to test for effects of the emotion regulation instructions (Gomez et al., 2015).

2. The *p* values for the two-tailed comparisons were as follows: difference PT-II versus CG, *p* < .001; difference PT-II versus GI, *p* = .033; difference PT-II versus GI-PT, *p* = .037.
