# Supplementary material for: The Cue-Response Mental Link: Its Critical Role in the Downregulation of Disgust by Perspective Taking Implementation Intention
Source: Psychol Rep. 2024 Oct 6;129(4):3101–22. doi: 10.1177/00332941241291030 (PMC13287364; doi:10.1177/00332941241291030)
Supplement: Supplemental Material - The Cue-Response Mental Link: Its Critical Role in the Downregulation of Disgust by Perspective Taking Implementation Intention [file sj-pdf-1-prx-10.1177_00332941241291030.pdf]

Supplementary Material of the paper “The cue-response mental link: its critical role in the downregulation of disgust by perspective taking implementation intention”

1 IAPS pictures

Source: Lang, P. J., Bradley, M. M., & Cuthbert, B. N. (2005). International affective picture system (IAPS): Digitized photographs, instruction manual and affective ratings. Technical Report A-6 Gainesville (FL): The Center for Research in Psychophysiology, University of Florida. The normative ratings are for females.

1.1 Practice pictures

**Affective category**	**IAPS number**	**Normative Valence**	**Normative Arousal**
Neutral	2104	4.42	3.22
Pleasant	2660	8.18	4.76
Disgusting	3150	1.98	6.94
Pleasant	4680	6.91	6.07
Neutral	7100	5.20	2.73
Pleasant	7280	7.73	4.33

1.2 Pictures of Set 1

**Affective category**	**IAPS number**	**Normative Valence**	**Normative Arousal**
Disgusting	3062	1.62	5.84
Disgusting	3064	1.15	7.30
Disgusting	3068	1.18	7.09
Disgusting	3069	1.32	7.33
Disgusting	3400	2.06	7.12
Neutral	2102	5.13	2.92
Neutral	2221	4.33	3.05
Neutral	7041	5.02	2.53
Neutral	7052	5.24	2.57
Neutral	7090	5.44	2.92
Pleasant	2057	8.39	4.73
Pleasant	4659	6.15	6.47
Pleasant	7230	7.35	5.27
Pleasant	7400	7.30	5.44
Pleasant	7480	7.00	4.02

1.3 Pictures of Set 2

Affective category	IAPS number	Normative Valence	Normative Arousal
Disgusting	3000	1.21	7.77
Disgusting	3010	1.29	7.44
Disgusting	3030	1.51	7.13
Disgusting	3051	2.06	6.00
Disgusting	3053	1.15	7.51
Disgusting	3060	1.66	7.34
Disgusting	3071	1.69	7.10
Disgusting	3080	1.33	7.61
Disgusting	3100	1.35	7.02
Disgusting	3102	1.22	7.15
Disgusting	3120	1.33	7.49
Disgusting	3130	1.26	7.39
Disgusting	3140	1.50	6.94
Disgusting	3550	1.98	6.14
Disgusting	9405	1.59	6.77
Neutral	2190	4.90	2.50
Neutral	2200	4.95	4.03
Neutral	2210	4.36	4.41
Neutral	2220	4.88	4.84
Neutral	2230	4.41	4.14
Neutral	2570	4.95	2.98
Neutral	7002	5.03	3.28
Neutral	7009	4.89	3.26
Neutral	7010	4.92	1.97
Neutral	7030	4.57	3.22
Neutral	7040	4.66	2.90
Neutral	7080	5.10	2.67
Neutral	7233	5.15	2.96
Neutral	7235	5.06	2.94
Neutral	9070	5.13	3.73
Pleasant	2070	8.50	4.84
Pleasant	2080	8.46	4.94
Pleasant	2160	8.16	5.03
Pleasant	2165	8.29	5.05
Pleasant	4651	5.15	5.71
Pleasant	4652	5.65	5.98
Pleasant	4653	6.04	5.68
Pleasant	4687	6.64	6.49
Pleasant	7200	7.77	4.85
Pleasant	7260	7.31	5.31
Pleasant	7270	7.77	5.85
Pleasant	7330	7.96	5.54
Pleasant	7350	7.11	4.67
Pleasant	7430	7.35	5.00
Pleasant	7470	7.18	4.72

2 SPSS Syntax

Final model for the outcome “valence rating”

```
MIXED Valence BY GROUP Set Picture_Category
  /CRITERIA=CIN(95) MXITER(100) MXSTEP(10) SCORING(1) SINGULAR(0.000000000001)
HCONVERGE(0,
    ABSOLUTE) LCONVERGE(0, ABSOLUTE) PCONVERGE(0.000001, ABSOLUTE)
/FIXED=GROUP Picture_Category Set GROUP*Picture_Category GROUP*Set
Set*Picture_Category Group*Set*Picture_Category| SSTYPE(3)
/METHOD=REML
/PRINT=SOLUTION TESTCOV G R
/RANDOM= Picture_Category Set*Picture_Category| SUBJECT(ID) COVTYPE(VC)
/RANDOM=INTERCEPT | SUBJECT(PictureNumber) COVTYPE(VC)
  /EMMEANS=TABLES(Group) COMPARE ADJ(LSD)
  /EMMEANS=TABLES(Picture_Category) COMPARE ADJ(LSD)
 /EMMEANS=TABLES(Set) COMPARE ADJ(LSD)
 /EMMEANS=TABLES(Group*Picture_Category) COMPARE (Group) ADJ(LSD)
 /EMMEANS=TABLES(Group*Picture_Category) COMPARE (Picture_Category) ADJ(LSD)
 /EMMEANS=TABLES(Group*Set) COMPARE (Group) ADJ(LSD)
 /EMMEANS=TABLES(Group*Set) COMPARE (Set) ADJ(LSD)
 /EMMEANS=TABLES(Set*Picture_Category) COMPARE (Picture_Category) ADJ(LSD)
 /EMMEANS=TABLES(Set*Picture_Category) COMPARE (Set) ADJ(LSD)
 /EMMEANS=TABLES(Group*Set*Picture_Category) COMPARE (Group) ADJ(LSD)
 /EMMEANS=TABLES(Group*Set*Picture_Category) COMPARE (Set) ADJ(LSD)
/TEST="GROUP IIAvsCG & Set1v2 at PictureCategory=3" GROUP*Set -1 1 0 0 0 0 1 -1
GROUP*Set*Picture_Category 0 0 -1 0 0 1 0 0 0 0 0 0 0 0 0 0 0 0 0 1 0 0 -1
/TEST="GROUP IIAvsGI & Set1v2 at PictureCategory=3" GROUP*Set 0 0 -1 1 0 0 1 -1
GROUP*Set*Picture_Category 0 0 0 0 0 0 0 0 -1 0 0 1 0 0 0 0 0 0 0 1 0 0 -1
/TEST="GROUP IIAvsGI-A & Set1v2 at PictureCategory=3" GROUP*Set 0 0 0 0 -1 1 1 -1
GROUP*Set*Picture_Category 0 0 0 0 0 0 0 0 0 0 0 0 0 0 -1 0 0 1 0 0 1 0 0 -1
/TEST="GROUP IIAvsCG & Set1v2 at PictureCategory=2" GROUP*Set -1 1 0 0 0 0 1 -1
GROUP*Set*Picture_Category 0 -1 0 0 1 0 0 0 0 0 0 0 0 0 0 0 0 0 1 0 0 -1 0
/TEST="GROUP IIAvsGI & Set1v2 at PictureCategory=2" GROUP*Set 0 0 -1 1 0 0 1 -1
GROUP*Set*Picture_Category 0 0 0 0 0 0 0 -1 0 0 1 0 0 0 0 0 0 0 1 0 0 -1 0
/TEST="GROUP IIAvsGI-A & Set1v2 at PictureCategory=2" GROUP*Set 0 0 0 0 -1 1 1 -1
GROUP*Set*Picture_Category 0 0 0 0 0 0 0 0 0 0 0 0 0 -1 0 0 1 0 0 1 0 0 -1 0
/TEST="GROUP IIAvsCG & Set1v2 at PictureCategory=1" GROUP*Set -1 1 0 0 0 0 1 -1
GROUP*Set*Picture_Category -1 0 0 1 0 0 0 0 0 0 0 0 0 0 0 0 0 1 0 0 -1 0 0
/TEST="GROUP IIAvsGI & Set1v2 at PictureCategory=1" GROUP*Set 0 0 -1 1 0 0 1 -1
GROUP*Set*Picture_Category 0 0 0 0 0 0 -1 0 0 1 0 0 0 0 0 0 0 1 0 0 -1 0 0
/TEST="GROUP IIAvsGI-A & Set1v2 at PictureCategory=1" GROUP*Set 0 0 0 0 -1 1 1 -1
GROUP*Set*Picture_Category 0 0 0 0 0 0 0 0 0 0 0 0 -1 0 0 1 0 0 1 0 0 -1 0 0.
```

Final model for the outcome “arousal rating”

```
MIXED Arousal BY GROUP Set Picture_Category
  /CRITERIA=CIN(95) MXITER(100) MXSTEP(10) SCORING(1) SINGULAR(0.000000000001)
HCONVERGE(0,
    ABSOLUTE) LCONVERGE(0, ABSOLUTE) PCONVERGE(0.000001, ABSOLUTE)
/FIXED=GROUP Picture_Category Set GROUP*Picture_Category GROUP*Set
Set*Picture_Category Group*Set*Picture_Category| SSTYPE(3)
/METHOD=REML
/PRINT=SOLUTION TESTCOV G R
/RANDOM=INTERCEPT Set Picture_Category| SUBJECT(ID) COVTYPE(VC)
/RANDOM=INTERCEPT | SUBJECT(PictureNumber) COVTYPE(VC)
  /EMMEANS=TABLES(Group) COMPARE ADJ(LSD)
  /EMMEANS=TABLES(Picture_Category) COMPARE ADJ(LSD)
 /EMMEANS=TABLES(Set) COMPARE ADJ(LSD)
 /EMMEANS=TABLES(Group*Picture_Category) COMPARE (Group) ADJ(LSD)
 /EMMEANS=TABLES(Group*Picture_Category) COMPARE (Picture_Category) ADJ(LSD)
 /EMMEANS=TABLES(Group*Set) COMPARE (Group) ADJ(LSD)
 /EMMEANS=TABLES(Group*Set) COMPARE (Set) ADJ(LSD)
 /EMMEANS=TABLES(Set*Picture_Category) COMPARE (Picture_Category) ADJ(LSD)
 /EMMEANS=TABLES(Set*Picture_Category) COMPARE (Set) ADJ(LSD)
 /EMMEANS=TABLES(Group*Set*Picture_Category) COMPARE (Group) ADJ(LSD)
```

```
 /EMMEANS=TABLES(Group*Set*Picture_Category) COMPARE (Set) ADJ(LSD)
/TEST="GROUP IIAvsCG & Set1v2 at PictureCategory=3" GROUP*Set -1 1 0 0 0 0 1 -1
GROUP*Set*Picture_Category 0 0 -1 0 0 1 0 0 0 0 0 0 0 0 0 0 0 0 0 0 1 0 0 -1
/TEST="GROUP IIAvsGI & Set1v2 at PictureCategory=3" GROUP*Set 0 0 -1 1 0 0 1 -1
GROUP*Set*Picture_Category 0 0 0 0 0 0 0 0 -1 0 0 1 0 0 0 0 0 0 0 0 1 0 0 -1
/TEST="GROUP IIAvsGI-A & Set1v2 at PictureCategory=3" GROUP*Set 0 0 0 0 -1 1 1 -1
GROUP*Set*Picture_Category 0 0 0 0 0 0 0 0 0 0 0 0 0 0 -1 0 0 1 0 0 1 0 0 -1
/TEST="GROUP IIAvsCG & Set1v2 at PictureCategory=2" GROUP*Set -1 1 0 0 0 0 1 -1
GROUP*Set*Picture_Category 0 -1 0 0 1 0 0 0 0 0 0 0 0 0 0 0 0 0 0 1 0 0 -1 0
/TEST="GROUP IIAvsGI & Set1v2 at PictureCategory=2" GROUP*Set 0 0 -1 1 0 0 1 -1
GROUP*Set*Picture_Category 0 0 0 0 0 0 0 -1 0 0 1 0 0 0 0 0 0 0 0 1 0 0 -1 0
/TEST="GROUP IIAvsGI-A & Set1v2 at PictureCategory=2" GROUP*Set 0 0 0 0 -1 1 1 -1
GROUP*Set*Picture_Category 0 0 0 0 0 0 0 0 0 0 0 0 0 -1 0 0 1 0 0 1 0 0 -1 0
/TEST="GROUP IIAvsCG & Set1v2 at PictureCategory=1" GROUP*Set -1 1 0 0 0 0 1 -1
GROUP*Set*Picture_Category -1 0 0 1 0 0 0 0 0 0 0 0 0 0 0 0 0 0 1 0 0 -1 0 0
/TEST="GROUP IIAvsGI & Set1v2 at PictureCategory=1" GROUP*Set 0 0 -1 1 0 0 1 -1
GROUP*Set*Picture_Category 0 0 0 0 0 0 -1 0 0 1 0 0 0 0 0 0 0 0 1 0 0 -1 0 0
/TEST="GROUP IIAvsGI-A & Set1v2 at PictureCategory=1" GROUP*Set 0 0 0 0 -1 1 1 -1
GROUP*Set*Picture_Category 0 0 0 0 0 0 0 0 0 0 0 0 -1 0 0 1 0 0 1 0 0 -1 0 0.
```

Final model for the outcome “disgust rating”

```
MIXED Disgust BY GROUP Set Picture_Category
  /CRITERIA=CIN(95) MXITER(100) MXSTEP(10) SCORING(1) SINGULAR(0.000000000001)
HCONVERGE(0,
    ABSOLUTE) LCONVERGE(0, ABSOLUTE) PCONVERGE(0.000001, ABSOLUTE)
  /FIXED=GROUP Picture_Category Set GROUP*Picture_Category GROUP*Set
Set*Picture_Category Group*Set*Picture_Category| SSTYPE(3)
  /METHOD=REML
  /PRINT=SOLUTION TESTCOV G R
  /RANDOM=INTERCEPT Picture_Category Picture_Category*Set | SUBJECT(ID) COVTYPE(VC)
  /RANDOM=INTERCEPT | SUBJECT(PictureNumber) COVTYPE(VC)
  /EMMEANS=TABLES(Group) COMPARE ADJ(LSD)
  /EMMEANS=TABLES(Picture_Category) COMPARE ADJ(LSD)
 /EMMEANS=TABLES(Set) COMPARE ADJ(LSD)
 /EMMEANS=TABLES(Group*Picture_Category) COMPARE (Group) ADJ(LSD)
 /EMMEANS=TABLES(Group*Picture_Category) COMPARE (Picture_Category) ADJ(LSD)
 /EMMEANS=TABLES(Group*Set) COMPARE (Group) ADJ(LSD)
 /EMMEANS=TABLES(Group*Set) COMPARE (Set) ADJ(LSD)
 /EMMEANS=TABLES(Set*Picture_Category) COMPARE (Picture_Category) ADJ(LSD)
 /EMMEANS=TABLES(Set*Picture_Category) COMPARE (Set) ADJ(LSD)
 /EMMEANS=TABLES(Group*Set*Picture_Category) COMPARE (Group) ADJ(LSD)
 /EMMEANS=TABLES(Group*Set*Picture_Category) COMPARE (Set) ADJ(LSD)
/TEST="GROUP IIAvsCG & Set1v2 at PictureCategory=3" GROUP*Set -1 1 0 0 0 0 1 -1
GROUP*Set*Picture_Category 0 0 -1 0 0 1 0 0 0 0 0 0 0 0 0 0 0 0 0 0 1 0 0 -1
/TEST="GROUP IIAvsGI & Set1v2 at PictureCategory=3" GROUP*Set 0 0 -1 1 0 0 1 -1
GROUP*Set*Picture_Category 0 0 0 0 0 0 0 0 -1 0 0 1 0 0 0 0 0 0 0 0 1 0 0 -1
/TEST="GROUP IIAvsGI-A & Set1v2 at PictureCategory=3" GROUP*Set 0 0 0 0 -1 1 1 -1
GROUP*Set*Picture_Category 0 0 0 0 0 0 0 0 0 0 0 0 0 0 -1 0 0 1 0 0 1 0 0 -1
/TEST="GROUP IIAvsCG & Set1v2 at PictureCategory=2" GROUP*Set -1 1 0 0 0 0 1 -1
GROUP*Set*Picture_Category 0 -1 0 0 1 0 0 0 0 0 0 0 0 0 0 0 0 0 0 1 0 0 -1 0
/TEST="GROUP IIAvsGI & Set1v2 at PictureCategory=2" GROUP*Set 0 0 -1 1 0 0 1 -1
GROUP*Set*Picture_Category 0 0 0 0 0 0 0 -1 0 0 1 0 0 0 0 0 0 0 0 1 0 0 -1 0
/TEST="GROUP IIAvsGI-A & Set1v2 at PictureCategory=2" GROUP*Set 0 0 0 0 -1 1 1 -1
GROUP*Set*Picture_Category 0 0 0 0 0 0 0 0 0 0 0 0 0 -1 0 0 1 0 0 1 0 0 -1 0
/TEST="GROUP IIAvsCG & Set1v2 at PictureCategory=1" GROUP*Set -1 1 0 0 0 0 1 -1
GROUP*Set*Picture_Category -1 0 0 1 0 0 0 0 0 0 0 0 0 0 0 0 0 0 1 0 0 -1 0 0
/TEST="GROUP IIAvsGI & Set1v2 at PictureCategory=1" GROUP*Set 0 0 -1 1 0 0 1 -1
GROUP*Set*Picture_Category 0 0 0 0 0 0 -1 0 0 1 0 0 0 0 0 0 0 0 1 0 0 -1 0 0
/TEST="GROUP IIAvsGI-A & Set1v2 at PictureCategory=1" GROUP*Set 0 0 0 0 -1 1 1 -1
GROUP*Set*Picture_Category 0 0 0 0 0 0 0 0 0 0 0 0 -1 0 0 1 0 0 1 0 0 -1 0 0.
```

Final model for the outcome “SCL”

```
MIXED SCL BY GROUP Set
  /CRITERIA=CIN(95) MXITER(100) MXSTEP(10) SCORING(1) SINGULAR(0.000000000001)
HCONVERGE(0,
    ABSOLUTE) LCONVERGE(0, ABSOLUTE) PCONVERGE(0.000001, ABSOLUTE)
/FIXED=GROUP Set GROUP*Set| SSTYPE(3)
/METHOD=REML
/PRINT=SOLUTION TESTCOV G R
/RANDOM=INTERCEPT| SUBJECT(ID) COVTYPE(VC)
  /EMMEANS=TABLES(Group) COMPARE ADJ(LSD)
 /EMMEANS=TABLES(Set) COMPARE ADJ(LSD)
 /EMMEANS=TABLES(Group*Set) COMPARE (Group) ADJ(LSD)
 /EMMEANS=TABLES(Group*Set) COMPARE (Set) ADJ(LSD).
```

3 Means and *SD*s (in parentheses) of valence rating, arousal rating, and disgust rating of the three picture categories (disgusting, neutral, pleasant). Note that these values are obtained by using all single ratings given to each picture by each participant. Ns used to obtain these means and *SD*s are as follows: n = 105 for Set 1 of CG, GI, PT-II; n = 115 for Set 1 of GI-PT; n = 315 for Set 2 of CG, GI, PT-II; n = 345 for Set 2 of GI-PT.

3.1 Means and *SD*s (in parentheses) of valence, arousal, and disgust ratings for the **disgusting pictures** of Set 1 and Set 2 for control (CG), goal intention (GI), goal intention with perspective taking (GI-PT), and goal intention with perspective taking implementation intention (PT-II)

	Valence		Arousal		Disgust	
	Set 1	Set 2	Set 1	Set 2	Set 1	Set 2
CG	1.54 (0.11)	1.98 (0.07)	6.72 (0.20)	5.81 (0.12)	7.54 (0.19)	6.73 (0.12)
GI	1.40 (0.08)	1.76 (0.06)	6.87 (0.19)	5.68 (0.12)	7.89 (0.15)	6.40 (0.13)
GI-PT	1.57 (0.10)	1.88 (0.06)	6.68 (0.22)	5.73 (0.11)	7.78 (0.18)	6.26 (0.12)
PT-II	1.34 (0.07)	1.93 (0.06)	7.07 (0.20)	5.78 (0.12)	8.05 (0.14)	5.77 (0.13)

3.2 Means and *SD*s (in parentheses) of valence, arousal, and disgust ratings for the **neutral pictures** of Set 1 and Set 2 for control (CG), goal intention (GI), goal intention with perspective taking (GI-PT), and goal intention with perspective taking implementation intention (PT-II)

	Valence		Arousal		Disgust	
	Set 1	Set 2	Set 1	Set 2	Set 1	Set 2

CG	5.46 (0.14)	5.04 (0.07)	2.34 (0.17)	2.50 (0.10)	1.19 (0.05)	1.57 (0.07)
GI	5.30 (0.12)	5.00 (0.07)	2.01 (0.14)	2.31 (0.10)	1.15 (0.05)	1.34 (0.05)
GI-PT	5.29 (0.11)	4.91 (0.06)	2.38 (0.17)	2.30 (0.10)	1.32 (0.08)	1.36 (0.05)
PT-II	5.39 (0.13)	4.83 (0.07)	2.73 (0.19)	2.97 (0.12)	1.43 (0.11)	1.53 (0.07)

3.3 Means and *SD*s (in parentheses) of valence, arousal, and disgust ratings for the **pleasant pictures** of Set 1 and Set 2 for control (CG), goal intention (GI), goal intention with perspective taking (GI-PT), and goal intention with perspective taking implementation intention (PT-II)

	Valence		Arousal		Disgust	
	Set 1	Set 2	Set 1	Set 2	Set 1	Set 2
CG	7.12 (0.12)	6.90 (0.08)	4.17 (0.19)	4.17 (0.11)	1.47 (0.12)	1.58 (0.08)
GI	7.39 (0.13)	7.05 (0.08)	4.24 (0.23)	3.94 (0.13)	1.30 (0.08)	1.26 (0.04)
GI-PT	7.25 (0.13)	6.81 (0.09)	4.30 (0.21)	4.04 (0.12)	1.49 (0.11)	1.62 (0.08)
PT-II	7.33 (0.13)	6.97 (0.09)	4.82 (0.23)	4.55 (0.13)	1.41 (0.10)	1.55 (0.07)

Sensitivity analyses

Statistics for the four-way interaction involving Experimental condition, Picture type, Picture set, and the specific variable Age, Disgust sensitivity, etc.

Valence rating

Four-way interaction with…	*F* statistics	*P* value
Age	$F(6, 245.69) = 1.29$	.26
Disgust sensitivity	$F(6, 244.74) = 0.82$	.56
Habitual use of cognitive reappraisal	$F(6, 244.71) = 0.75$	.61
Commitment	$F(6, 244.48) = 1.25$	.28
Control of negative feelings	$F(6, 244.42) = 0.37$	.90
Reappraisal of unpleasant pictures	$F(6, 244.32) = 0.85$	.54
Ignoring unpleasant pictures	$F(6, 245.21) = 1.83$	.094
Difficulty controlling negative feelings	$F(6, 244.63) = 0.69$	.66
Usefulness of instructions	$F(6, 244.69) = 1.52$	.17
Success in reaching instruction’s goal	$F(6, 244.83) = 0.91$	.48
Presentation Order	$F(24, 206.74) = 1.00$	.47

Arousal rating

Four-way interaction with…	*F* statistics	*P* value
Age	*F*(6, 238.31) = 0.43	.85
Disgust sensitivity	*F*(6, 4746.04) = 0.53	.78
Habitual use of cognitive reappraisal	*F*(6, 4878) = 0.28	.95
Commitment	*F*(6, 238) = 0.43	.86
Control of negative feelings	*F*(6, 4746.29) = 0.39	.89
Reappraisal of unpleasant pictures	*F*(6, 4753.21) = 1.82	.091
Ignoring unpleasant pictures	*F*(6, 4745.84) = 1.49	.18
Difficulty controlling negative feelings	*F*(6, 4746) = 0.59	.74
Usefulness of instructions	*F*(6, 4745.78) = 0.52	.79
Success in reaching instruction's goal	*F*(6, 4763.95) = 1.67	.12
Presentation Order	*F*(24, 4721.56) = 1.51	.051

Disgust rating

Four-way interaction with…	*F* statistics	*P* value
Age	*F*(6, 236.03) = 1.46	.19
Disgust sensitivity	*F*(6, 236.23) = 1.15	.34
Habitual use of cognitive reappraisal	*F*(6, 235.95) = 0.98	.44
Commitment	*F*(6, 236.01) = 0.55	.77
Control of negative feelings	*F*(6, 236.16) = 0.66	.68
Reappraisal of unpleasant pictures	*F*(6, 236.07) = 2.36	.031
Ignoring unpleasant pictures	*F*(6, 235.98) = 0.45	.84
Difficulty controlling negative feelings	*F*(6, 236.76) = 0.62	.71
Usefulness of instructions	*F*(6, 236.11) = 1.04	.40
Success in reaching instruction's goal	*F*(6, 414.72) = 0.17	.98
Presentation Order	*F*(24, 199.90) = 1.14	.30

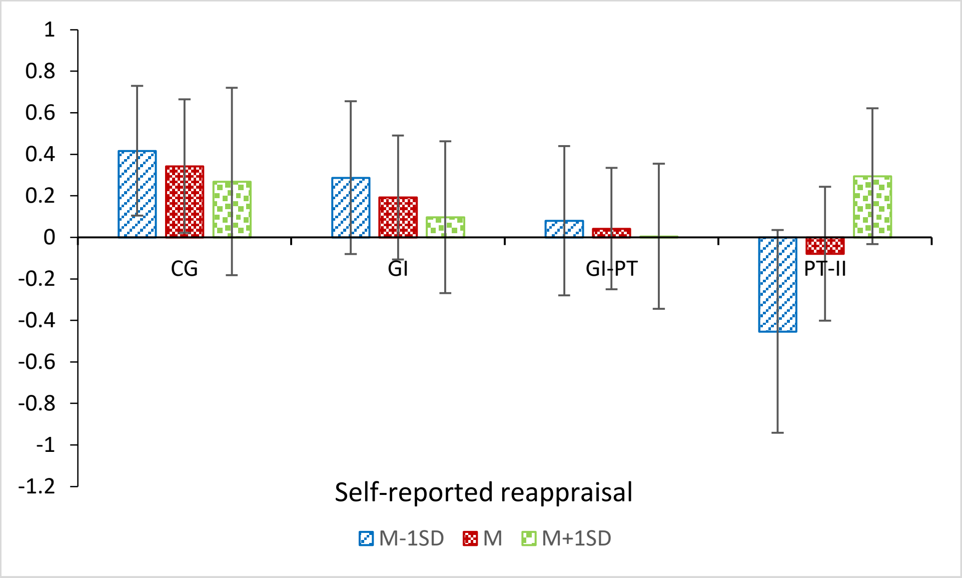

Fig. S1. Estimated mean changes from Set 1 to Set 2 in disgust rating of the **neutral pictures** for the four experimental conditions as a function of self-reported conscious attempt to reappraise the disgusting pictures (question "How much did you try to think about the unpleasant pictures in a way that decreased your emotion?", rated on a 1-9 scale). *M*-1*SD*, *M*, and *M*+1*SD* correspond to 3.44, 5.97, and 8.49, respectively. Bars represent *SE*s. CG = control group (no emotion regulation instructions); GI = goal intention group; GI-PT = goal intention with perspective taking strategy group; PT-II = goal intention with perspective taking implementation intention group.

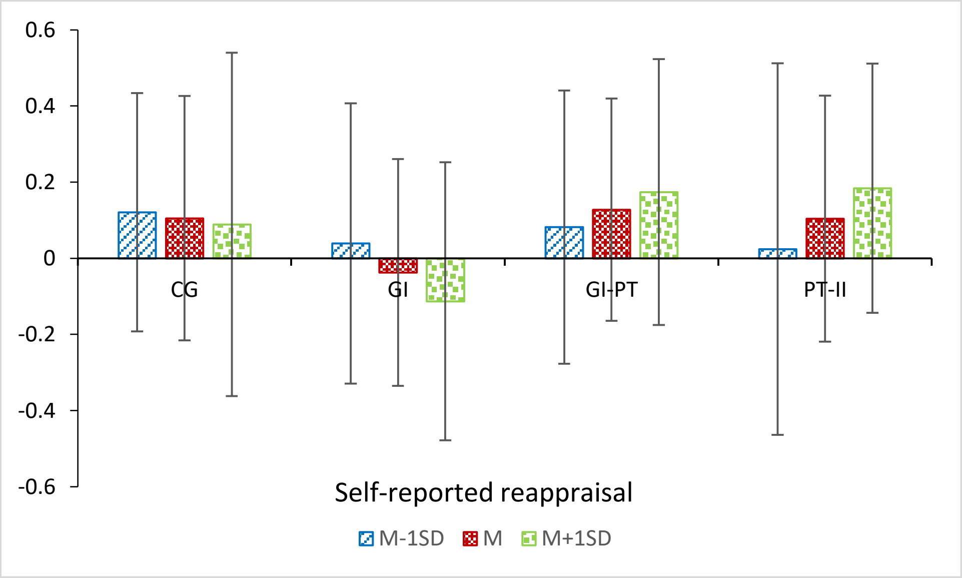

Fig. S2. Estimated mean changes from Set 1 to Set 2 in disgust rating of the **pleasant pictures** for the four experimental conditions as a function of self-reported conscious attempt to reappraise the disgusting pictures (question "How much did you try to think about the unpleasant pictures in a way that decreased your emotion?", rated on a 1-9 scale). *M*-1*SD*, *M*, and *M*+1*SD* correspond to 3.44, 5.97, and 8.49, respectively. Bars represent *SE*s. CG = control group (no emotion regulation instructions); GI = goal intention group; GI-PT = goal intention with perspective taking strategy group; PT-II = goal intention with perspective taking implementation intention group.

Comparisons between participants of this study and participants of Gomez et al. (2015)

We compared the three groups CG, GI, and PT-II that were in both studies for age, disgust sensitivity, habitual use of cognitive reappraisal, and the four questions “How committed did you feel to the regulation intention/instructions?’’, ‘‘How much did you try to control your negative feelings when looking at the unpleasant pictures?’’, ‘‘How much did you try to think about the unpleasant pictures in a way that decreased your emotion?’’, and ‘‘How much did you try to ignore the unpleasant pictures?’’, For each variable, we tested the main effects of Study and Experimental condition and their interaction.

Age. The effects of Study, $F(1, 117) = 0.92$, $p = .34$, Experimental condition, $F(2, 117) = 1.20$, $p = .30$, and interaction Study by Experimental condition, $F(2, 117) = 0.02$, $p = .98$, were not significant.

Disgust sensitivity. The effects of Study, $F(1, 117) = 0.84$, $p = .36$, Experimental condition, $F(2, 117) = 0.42$, $p = .65$, and interaction Study by Experimental condition, $F(2, 117) = 1.66$, $p = .19$, were not significant.

Habitual use of cognitive reappraisal. The effects of Study, $F(1, 117) = 0.57$, $p = .45$, Experimental condition, $F(2, 117) = 0.03$, $p = .97$, and interaction Study by Experimental condition, $F(2, 117) = 2.97$, $p = .055$, were not significant.

How committed did you feel to the regulation intention/instructions? The effects of Study, $F(1, 117) = 2.54$, $p = .11$, Experimental condition, $F(2, 117) = 0.65$, $p = .52$, and interaction Study by Experimental condition, $F(2, 117) = 0.25$, $p = .78$, were not significant.

How much did you try to control your negative feelings when looking at the unpleasant pictures? The effects of Study, $F(1, 117) = 0.33$, $p = .57$, and interaction Study by Experimental condition, $F(2, 117) = 0.47$, $p = .63$, were not significant. The effect of Experimental condition was significant, $F(2, 117) = 21.64$, $p < .001$.

How much did you try to think about the unpleasant pictures in a way that decreased your emotion? The effect of Study was significant, $F(1, 117) = 5.59$, $p = .020$. Participants of the present study had a higher mean ($M = 5.97$, $SE = 0.27$) than participants of Gomez et al.' (2015) study ($M = 5.16$, $SE = 0.28$). The effect of Experimental condition was significant, $F(2, 117) = 9.03$, $p < .001$. The effect of interaction Study by Experimental condition was not significant, $F(2, 117) = 0.44$, $p = .65$.

How much did you try to ignore the unpleasant pictures? The effects of Study, $F(1, 117) = 2.14$, $p = .15$, Experimental condition, $F(2, 117) = 2.26$, $p = .11$, and interaction Study by Experimental condition, $F(2, 117) = 0.93$, $p = .40$, were not significant.
